# Study on the Compatibility of SBR and Asphalt Base Based on Molecular Simulation

**DOI:** 10.3390/ma17051175

**Published:** 2024-03-02

**Authors:** Xiaolei Jiao, Dandan Huang, Song Zhao, Jian Ouyang

**Affiliations:** 1 Tianjin Highway Development Service Center, Tianjin 300170, China; jxl9132@163.com; 2 Tianjin GUOTENG Highway Consultation & PROJECT Management CO., Ltd., Tianjin 300170, China; huangdandan8674@163.com; 3 School of Civil Engineering and Transportation, Northeast Forestry University, Harbin 150040, China; zsnefu0719@163.com; 4 School of Civil Engineering and Architecture, Hainan University, Haikou 570228, China

**Keywords:** molecular dynamics, compatibility, solubility parameters, modified asphalt

## Abstract

In the field of highway construction, the application of styrene–butadiene rubber (SBR)-modified asphalt has gained popularity across different levels of road surfaces. A crucial aspect in ensuring the efficacy of this modification lies in the compatibility between SBR and the matrix asphalt. To address this, the current study utilizes molecular dynamics simulation as a technique. By establishing a model for the SBR-modified asphalt mixture, the research quantifies the compatibility level between the SBR modifier and the asphalt. The aim is to uncover the underlying mechanisms of compatibility between the SBR modifier and the base asphalt, ultimately contributing to the improvement of the storage stability of SBR-modified asphalt, which holds significant importance. The investigation began with the creation of models for both the base asphalt and the SBR modifier. A model for the SBR-modified asphalt blending system was then formulated based on these initial models. After undergoing geometry optimization and annealing procedures, the model attained its lowest energy state, providing a reliable basis for examining the performance of SBR-modified asphalt. The study proceeded to calculate solubility parameters and interaction energies of the system to evaluate the compatibility between the SBR modifier and the base asphalt at various temperatures. The analysis of these parameters shed light on the compatibility mechanism between the two components. Notably, it was found that at a temperature of 160 ℃, the compatibility was significantly enhanced. The findings were further corroborated through scanning electron microscope and rheological tests. The outcomes of this research offer theoretical guidance for the application of SBR-modified asphalt.

## 1. Introduction

The modification of base asphalt has been extensively implemented in practical applications across various countries for many years. Styrene–butadiene–styrene (SBS) is predominantly employed for this purpose, primarily due to its ability to enhance multiple properties of the base asphalt, resulting in superior modification effects [1]. SBS is a block copolymer composed of styrene and butadiene monomers, exhibiting characteristics of both plastics and rubbers. It demonstrates compatibility with water, weak acids, and alkalis and possesses outstanding tensile strength, a high surface friction coefficient, commendable low-temperature performance, excellent electrical properties, and good processability. These attributes have led to SBS becoming the most widely consumed thermoplastic elastomer. While SBS is often utilized for high-grade highway asphalt pavements due to its superior modification effects, its high cost renders it less suitable for ordinary road applications. Conversely, SBR, a widely used synthetic rubber in industrial production, presents a more cost-effective alternative [2]. SBR is a random copolymer of butadiene and styrene, known for its well-rounded properties. When blended with asphalt, it forms a rubber network that enhances the asphalt’s characteristics [3]. Although the performance of SBR-modified asphalt may not match that of SBS-modified asphalt, it still offers significant improvements. Therefore, investigating the compatibility between SBR and asphalt is essential.

Molecular dynamics simulation, acclaimed for its microscopic precision, is extensively utilized to predict the macroscopic physical attributes of asphalt and lay a theoretical groundwork for studies on asphalt performance [4,5,6,7]. This technique is predominantly applied to examine a range of aspects, including asphalt diffusion behavior, compatibility between asphalt and modifiers, and the adhesion characterization between asphalt and aggregates. For example, Guo et al. employed a solubility parameter and binding energy as evaluation criteria to analyze the compatibility of matrix asphalt and rubber-modified asphalt, thereby identifying the optimal dosage of rubber-modified asphalt modifier via molecular dynamics simulation [8]. Yu et al. investigated the compatibility of epoxy and matrix asphalt using a solubility parameter and interaction energy as assessment metrics, uncovering that the incorporation of biobased epoxidized soybean oil improved the compatibility of the modified asphalt system [9]. Bhasin et al. utilized molecular dynamics simulations to study the relationship between the asphalt chain length, branched chains, and the asphalt self-diffusion coefficient, thus enhancing the understanding of the interplay between the asphalt molecular structure, self-diffusion coefficient, and self-healing performance [10]. Furthermore, Ding et al. explored the diffusion mechanism between new and aged asphalt, finding that asphalt macromolecules, particularly asphaltene, played a crucial role in asphalt’s sensitivity to temperature and aging. They also discovered that the addition of a regenerant to aged asphalt facilitated mutual diffusion between new and old asphalt, thereby improving recycling efficiency [11].

During the production of modified asphalt, the direct inclusion of modifiers frequently leads to segregation. This is attributed to the differences in structural and external characteristics between the modifier and the asphalt, which hinder uniform dispersion within the asphalt [12,13]. Thermodynamically, modifiers and asphalt can only achieve limited compatibility, resulting in a partially compatible two-phase system. Therefore, ensuring compatibility between these components is vital for creating high-quality modified asphalt, highlighting the importance of assessing their compatibility [14]. Currently, methods for evaluating the compatibility of modified asphalt from both microscopic and macroscopic perspectives include the segregation method [15], glass transition temperature analysis [16], electron microscopy [17], infrared spectroscopy [18], and the Cole–Cole diagram method [19], among others. Each method provides distinct insights into the compatibility between modifiers and asphalt, aiding in the refinement of modified asphalt production techniques.

The aim of this paper is to establish the calculation parameters and fundamental models used in molecular dynamics simulations, building on previous research. The focus is on developing molecular models for both the SBR modifier and asphalt. The main goal is to explore the compatibility between the SBR modifier and matrix asphalt at different temperatures. By examining the solubility parameters and interaction energy within the modified asphalt blending system, we can gain a deeper understanding of the compatibility mechanisms between the asphalt and modifiers. Microscopic insights into the compatibility mechanism between SBR and the matrix asphalt are obtained through scanning electron microscopy (SEM) testing. Additionally, the compatibility is evaluated from a macroscopic perspective through rheological testing. Moreover, by comparing the simulation results with experimental data, the reliability of the findings can be enhanced, providing strong support for experimental design and results interpretation. In conclusion, molecular simulation offers significant innovative potential in understanding the compatibility between asphalt and SBR modifiers. It serves as an important tool for providing theoretical guidance and technical support for the design and engineering application of modified asphalt.

### 1.1. Construction of Molecular Models

#### 1.1.1. Asphalt

**Figure 1:**
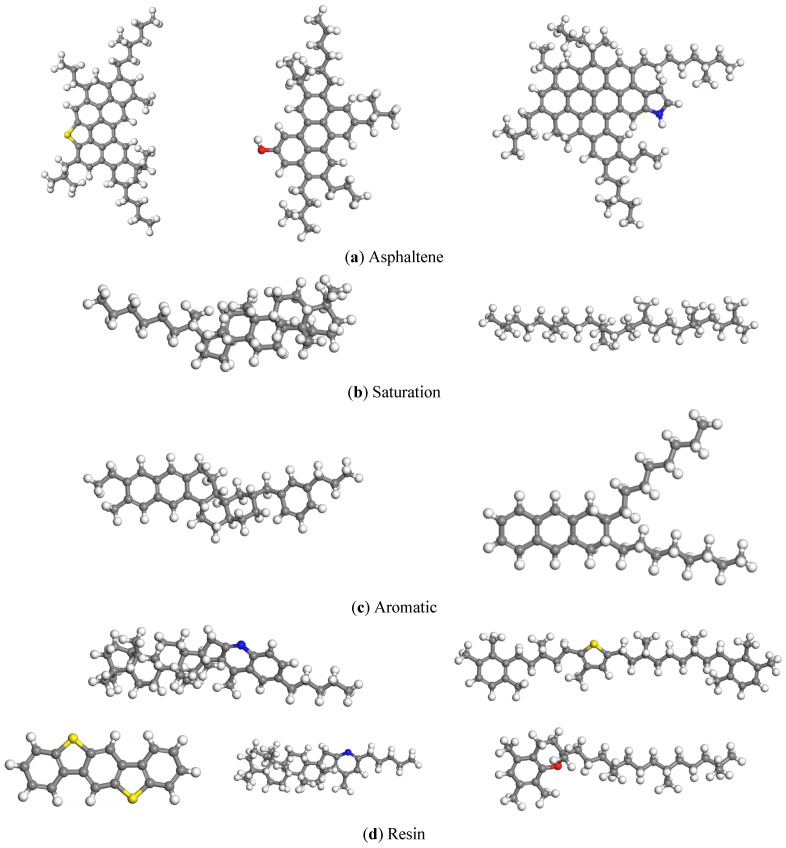
Asphalt molecular component structure formula. (Yellow represents sulfur atoms, red represents oxygen atoms, blue represents nitrogen atoms, gray represents carbon atoms, and white represents hydrogen atoms).

In the realm of molecular simulation for asphalt molecular modeling, two primary techniques are employed: the average molecular structure method and the assembly method. The former emphasizes simulation efficiency and the conservation of computational resources, albeit at the expense of neglecting interactions between different components within the asphalt structure. The latter, however, can be subdivided into two strategies: one involves selecting the average structure of each asphalt component, while the other involves choosing representative molecules from each component to represent the asphalt structure. Chu et al. [20] found that the four-component model constructed using the representative molecule approach demonstrated a higher accuracy and suitability for molecular dynamics simulations. Consequently, this study adopts the approach of constructing a representative molecular model for each asphalt component to develop a matrix asphalt molecular model. The matrix asphalt molecular model employed in this research is derived from the asphalt model proposed by Li et al. [21], which includes saturated components, aromatic components, resins, and asphaltenes, with each component consisting of multiple molecules, totaling 12 molecules. The asphalt molecular model was then assembled based on the proportions of the Panjin 90 matrix asphalt (produced by Northern Asphalt in Panjin, Liaoning Province, China) components used in this study, as outlined in Table 1. The structural representation of each component is illustrated in Figure 1.

#### 1.1.2. SBR Modifier

**Figure 2:**
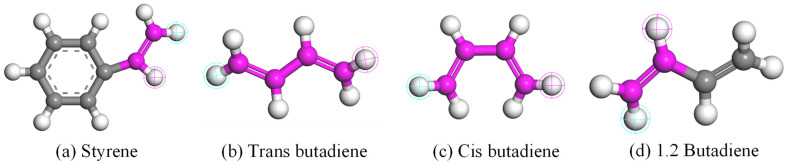
Molecular formula of modifier. (The atoms wrapped in the light blue net are the head of the chain structure, and the atoms wrapped in the pink net are the tail of the chain structure).

Styrene–butadiene rubber is a synthetic rubber produced through the copolymerization of butadiene and styrene. Its chemical formula is commonly denoted as (C8H8)_x_(C4H6)_y_, where ‘x’ and ‘y’ signify the molar ratio of styrene and butadiene monomers, typically falling within the range of 20:80 to 30:70. The composition ratio of SBR is outlined in Table 2, with the structural formula of each component depicted in Figure 2.

**Figure 3:**
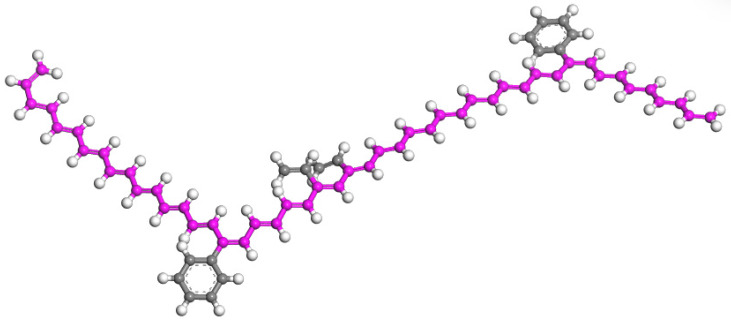
SBR single chain molecule. (The pink color is the aggregation path).

Studies have shown that a two-phase continuous state occurs between SBR and matrix asphalt when the SBR content is between 4% and 5%. For simplification purposes, we will consider the degree of polymerization for SBR to be 1 (indicating that the SBR molecule consists of a single repeating unit). Based on the structural representation shown in Figure 2, Build Polymer Plate was used to create an SBR block random copolymer with a degree of polymerization of 1, as shown in Figure 3.

### 1.2. Building a Crystal Cell Model

**Figure 4:**
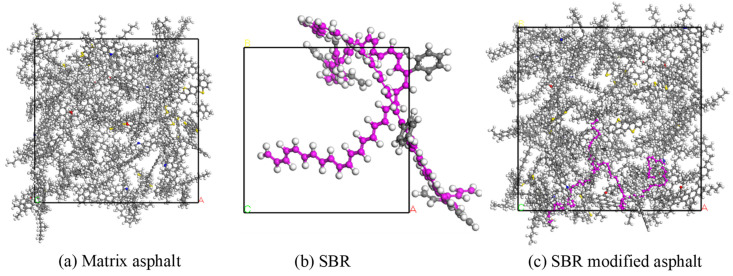
Crystal cell model of asphalt and modifier.

Employing the molecular quantities outlined in Table 1 and Table 2 for the matrix asphalt and modifier models, respectively, the construction function within the amorphous cell module was utilized to create amorphous crystal cells for both the matrix asphalt and modifier. These cells were initialized at a density of 0.4 g/cm^3^ to avoid entanglement or overlapping of molecular chains. For the SBR-modified asphalt, SBR single chains were integrated into the matrix asphalt at a molar mass of 5%. The molecular models for the three crystal cell configurations are illustrated in Figure 4.

### 1.3. Molecular Model Optimization and Kinetic Operations

The geometry optimization function of the forcite module in Materials Studio 8.0 software is used to minimize the model energy. The maximum number of iterations is 20,000, and the force field is COMPASS. Throughout this process, the model’s energy gradually approaches its minimum and can achieve convergence. However, geometric optimization solely guarantees the lowest local energy for the molecular structure. To attain the configuration with the lowest global energy, annealing is necessary to fully reveal the model’s internal structure at elevated temperatures and eliminate any unreasonable structures. Given that this study employs periodic boundary conditions to construct a three-dimensional model, the Andersen temperature control method and the Berendsen air pressure method are chosen for system relaxation. In the NPT system, the initial temperature is set at 298 K, raised to 798 K, then lowered back to 298 K, with five annealing cycles conducted. Post-annealing, the system energy stabilizes, and any local irrational structures in the model are eradicated. Subsequently, a 100 ps dynamic calculation is carried out on the molecular model under the NPT system at room temperature (25 °C) and a pressure of 1 standard atmosphere (0.0001 Gpa). To ensure comprehensive software simulation performance, this study opts for a 1 fs step size, ultimately yielding a reasonable and stable molecular model.

### 1.4. Validation of Molecular Models

**Figure 5:**
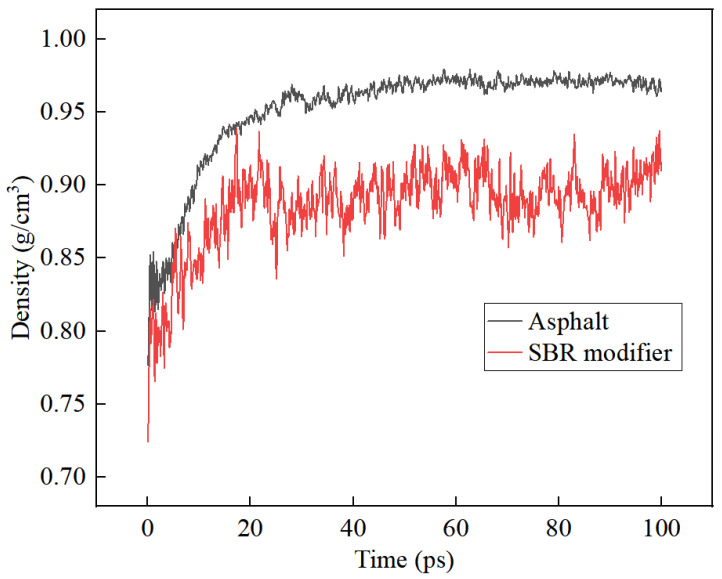
Variation in density of matrix asphalt with modifier.

The density curves for the asphalt model and the SBR modifier model, as obtained from the calculations under the conditions described in Section 1.3, are depicted in Figure 5. With increasing time steps, the curves gradually reach a stable state, exhibiting fluctuations within a range of no more than 5%. The density curves for matrix asphalt molecules stabilize around 0.98 g/cm^3^, while those for SBR modifier molecules stabilize at 0.93 g/cm^3^. The actual densities of matrix asphalt and the SBR modifier are reported to be 1.00 g/cm^3^ [22] and 0.94 g/cm^3^ [23], respectively, which are in close agreement with the simulation results. Consequently, it can be inferred that the molecular model of matrix asphalt and the molecular model of SBR modifier constructed in this study possess high applicability.

## 2. Compatibility Study of SBR with Asphalt Based on Molecular Simulation

### 2.1. Solubility Parameters

#### 2.1.1. Simulation Methods

Molecular dynamics simulations were conducted on models of matrix asphalt and the SBR modifier. Due to the fact that modified asphalt is produced at a higher heating temperature compared to matrix asphalt, kinetic equilibrium simulations of 100 ps were performed under the NVT system at temperatures of 140 °C, 150 °C, 160 °C, 170 °C, 180 °C, and 190 °C. This was performed to ensure the acquisition of more precise data for analysis. Meters of the SBR modifier and matrix asphalt at different temperatures. Finally, the cohesive energy density function was utilized to calculate the trajectory files obtained from the above temperatures to obtain the cohesive energy density as well as solubility parameters.

#### 2.1.2. Principle of Solubility Parameter Calculation

The concept of cohesive energy density was first proposed by Hildebrand in the context of blending theory, which uses cohesion energy to estimate the energy change when mixing two substances [24]. At the same time, the theory introduced the solubility parameter as a measure to assess the compatibility of two materials, especially non-polar polymers. The solubility parameter (*δ*) is defined as the square root of the cohesive energy density and acts as an indicator of molecular interaction properties. Therefore, the relationship between the solubility parameter and the cohesive energy density can be described by Equation (1).

(1) $$\delta =\sqrt{CED}$$

where *δ* is the solubility parameter ((J/cm^3^)^1/2^); *CED* is the cohesive energy density (J/cm^3^).

In the molecular dynamics (MD) simulation, the cohesive energy density of the materials is determined using the cohesive energy density panel in the MS 8.0 software, which facilitates the calculation of the solubility parameter (*δ*) of the materials for evaluating the compatibility of the polymers. When there is an absence of strong polar groups or hydrogen bonds between the molecules of the two materials, compatibility is achieved if the difference in the solubility parameter is minimal. Therefore, in this study, the difference in the solubility parameter is selected as an index to assess the degree of miscibility between the SBR modifier and matrix asphalt.

#### 2.1.3. Results and Discussion

**Figure 6:**
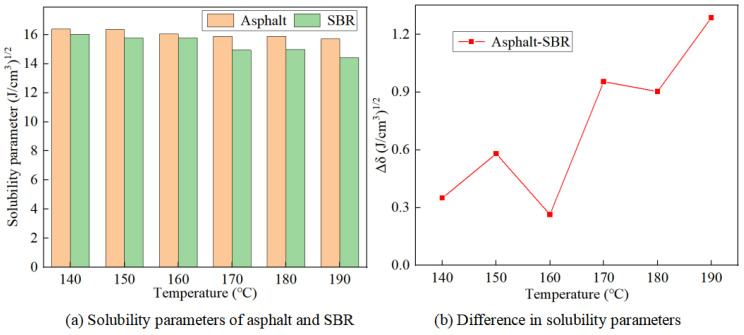
Calculated solubility parameters of asphalt and SBR.

Figure 6a illustrates the trend of solubility parameters of asphalt and SBR molecules with temperature, while Figure 6b illustrates the trend of solubility difference between the asphalt and SBR molecules with temperature.

With the rise in temperature, the molecular motion of both the matrix asphalt and SBR modifier intensifies, leading to an increase in the kinetic energy between molecules. As a result, the speed of molecular diffusion also increases along with the kinetic energy, causing the volume to expand. This expansion in volume leads to a greater separation between the molecules of the matrix asphalt and SBR modifier, as well as between atoms. When the distance surpasses the equilibrium distance, the nature of the intermolecular forces shifts from repulsive to attractive. Therefore, as the spacing between molecules widens, negative work is performed within the molecules of both the matrix asphalt and SBR modifier models, leading to a decrease in the bonding energies between molecules. In simpler terms, the bonding energy becomes more negative as the spacing between molecules increases.

With the increase in temperature, both the kinetic and potential energies of the matrix asphalt and SBR modifier rise, resulting in a higher total energy of the system. It is important to note that the intramolecular energy remains relatively stable during molecular dynamics simulations of asphalt mixtures [25]. This means that the intramolecular energies of the matrix asphalt and SBR modifier are consistent across various temperatures. The rise in total system energy is accompanied by a decrease in cohesive energy and an increase in volume. As a result, the cohesive energy densities of both the matrix asphalt and SBR modifier decrease, leading to a reduction in the solubility parameters of both the asphalt and SBR modifier.

The minimal disparity in solubility parameters between the matrix asphalt and SBR modifiers at 160 °C could be attributed to the similarities and differences in their molecular structures. Matrix asphalt is a complex blend of hydrocarbons, primarily composed of hydrocarbon chain polymers, while SBR modifiers are elastomers formed from the copolymerization of styrene and butadiene. The molecular structures of these two materials exhibit significant differences in their chemical composition and macroscopic properties, leading to variations in the simulated values and rates of change in their solubility parameters. At varying temperatures, the interaction between matrix asphalt and the SBR modifier is influenced by temperature, causing fluctuations in the differences between their solubility parameters, with a peak value at a specific temperature point. According to compatibility theory, the interaction between the matrix asphalt and SBR modifier may be optimal at 160 °C, as their molecular structures or chemical properties are more similar at this temperature, reducing mutual repulsion and enhancing their compatibility.

### 2.2. Interaction Energy

#### 2.2.1. Principle of Interaction Energy

The intermolecular interaction energy (*E_Inter_*) can quantitatively characterize the strength of intermolecular interactions to predict the mixing ability and compatibility of the two materials in the blended system [26], and the interaction energy can be characterized by Equation (2).

(2) $$E_{Inter}=E_{ab}-(E_{a}+E_{b})$$

where *E_ab_* is the total energy of an *a*, *b* co-mingled system (kJ/mol); *E_a_, E_b_* are the average single-point potential energy of an *a*, *b* system (kJ/mol).

When the system of intermolecular interaction energy is greater, the system of intermolecular interaction force is stronger, and the higher the stability of the system, the greater the performance of good compatibility. When the interaction energy is positive, it means that the SBR modifier and matrix asphalt exhibit mutual repulsion; when there is a negative value, it means that the SBR modifier and matrix asphalt exhibit mutual attraction.

As the SBR modifier and matrix asphalt are compatible only on the physical level of co-mingling, this paper will not consider the energy within the molecule. The total potential energy between molecules is mainly composed of van der Waals potential energy and electrostatic potential energy, so this paper will choose the total molecular potential energy (E_p_), van der Waals potential energy (E_v_), and electrostatic potential energy (E_e_) to characterize the interaction between molecules within the SBR-modified asphalt blending system, when the energy of these types of interactions is large enough to show that the interaction between the SBR-modified asphalt blending system is stronger, and the molecules of the SBR modifier and matrix asphalt are not easily separated. If the SBR modifier and matrix asphalt molecules are not easy to separate or destroy, then the system shows a good compatibility.

#### 2.2.2. Simulation Methods

Molecular dynamics simulation calculations were performed on the SBR-modified asphalt blend system model. The 100 ps kinetic flat calculation under the NVT ensemble was also performed at 140 °C, 150 °C, 160 °C, 170 °C, 180 °C, and 190 °C to achieve a full mixing process of the SBR modifier and matrix asphalt. Then, the dynamic calculation of 100 ps is continued under the NPT ensemble to reach a stable state. Finally, the stable configurations in the last three steps are selected, and the potential energies of different systems at different temperatures are calculated through the energy program in the software.

#### 2.2.3. Results and Discussion

According to the kinetic calculation results, the total potential energy (E_p_), van der Waals potential energy (E_v_), and electrostatic potential energy (E_e_) of the modified asphalt, matrix asphalt, and modifier were obtained. The average value of the operation results is shown in Table 3.

As indicated in Table 3, the total potential energy and van der Waals potential energy of the three molecular systems comprising base asphalt, the SBR modifier, and modified asphalt escalate with the rise in temperature, whereas the electrostatic potential energy exhibits fluctuations with the increasing temperature. This phenomenon occurs because, as the temperature increases, the system is heated, leading to a continuous rise in internal thermal energy and consequently, an increase in total energy. This thermal energy is then transformed into molecular potential energy and kinetic energy, resulting in the observed increase in total potential energy and kinetic energy.

As the kinetic energy rises, the volume of SBR-modified asphalt expands, leading to an increase in the distance between molecules in the system. However, since van der Waals forces act as attractive forces, their potential energy in the SBR-modified asphalt mixing system continues to increase as the distance between molecules grows, resulting in negative work by van der Waals forces. Electrostatic forces, being conservative, depend solely on the initial and final positions of the atoms, not on the path taken. The electrostatic potential energy is proportional to the product of the charges between the atoms and is influenced by the distance between them. Although the volume of the SBR-modified asphalt and the distance between atoms are increasing, the rate of increase is diminishing. Consequently, the electrostatic potential energy of the system fluctuates as the temperature increases.

**Figure 7:**
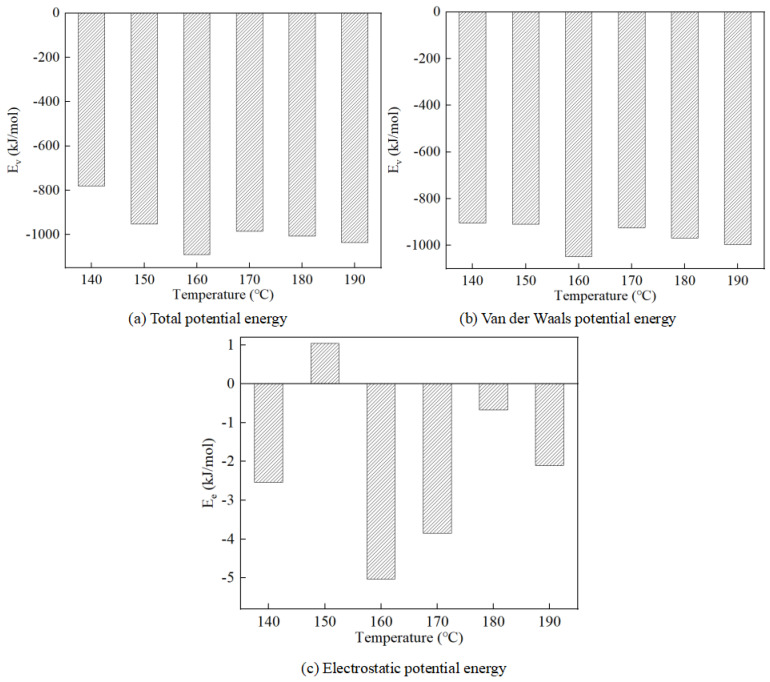
Interaction energy between matrix asphalt and SBR modifier at different temperatures.

The total potential energy, van der Waals potential energy, and electrostatic potential energy between the molecules of the matrix asphalt and SBR modifier in the SBR-modified asphalt mixing system were calculated using Equation (2), with the results presented in Figure 7.

The interaction energy between the SBR modifier and matrix asphalt is negative, indicating that the interaction between the molecules of the matrix asphalt and the SBR modifier in the SBR-modified asphalt system is characterized by attraction. Since the van der Waals force is an attractive force, it can be deduced that the interaction between the base asphalt and the SBR modifier is predominantly governed by van der Waals forces. Consequently, in the modified asphalt system, the interaction between the SBR modifier and the base asphalt molecules is primarily driven by van der Waals forces.

In the SBR-modified asphalt system, the interaction energy peaks at 160 °C, indicating that the force of interaction between the SBR modifier and the matrix asphalt is the strongest at this temperature within the mixed system. This phenomenon occurs because, with rising temperature, the kinetic energy of the molecules increases, leading to more vigorous motion between them. This enhanced motion facilitates the molecules’ escape from each other’s interaction range, thereby increasing the average distance between them. Consequently, interactions that seem repulsive at lower temperatures gradually transform into attraction as the temperature rises. Hence, the interaction between the SBR modifier and the matrix asphalt intensifies and reaches its maximum value at 160 °C.

At this temperature, the attraction between the SBR modifier molecules and the matrix asphalt molecules is the largest, while the repulsion is the smallest, so they ultimately exhibit a strong interaction. Therefore, at 160 °C, the intermolecular interaction in the SBR-modified asphalt system reaches an equilibrium state, making the system more stable and compatible. This is consistent with simulation results based on differences in solubility parameters.

## 3. SBR and Asphalt Compatibility Test Verification

### 3.1. Preparation of SBR-Modified Asphalt

In this paper, Panjin 90# matrix asphalt was selected to prepare modified asphalt, and its main technical indicators are shown in Table 4.

The SBR modifier used in this paper is a synthetic rubber produced in Panjin, Liaoning Province, used to modify asphalt, and the technical specifications are shown in Table 5.

In this paper, we only control the difference in shear temperature, respectively, 150 °C, 160 °C, 170 °C, and 180 °C, and the rest of the preparation conditions are the same. The preparation process of the specific SBR-modified asphalt is as follows.

The procedure entails setting the oven temperature to 140 °C and placing four 500 g batches of matrix asphalt into the oven for dehydration. Concurrently, the corresponding amount of SBR modifier is weighed. With the matrix asphalt temperature maintained at 140 °C, it is transferred to a shear apparatus. The pre-weighed SBR modifier is gradually added to the matrix asphalt, with continuous stirring using a glass rod for 15 min to ensure thorough mixing. The shear apparatus is operated at a rotation speed of 3000 r/min for 1 h. After shearing, the SBR-modified asphalt is placed back in the 140 °C oven for dissolution, with a duration of 1 h. Once the dissolution process is complete, the SBR-modified asphalt is cooled to room temperature, allowed to rest, and then subjected to related tests for modified asphalt.

### 3.2. Microscopic Morphology Analysis of Modified Asphalt

**Figure 8:**
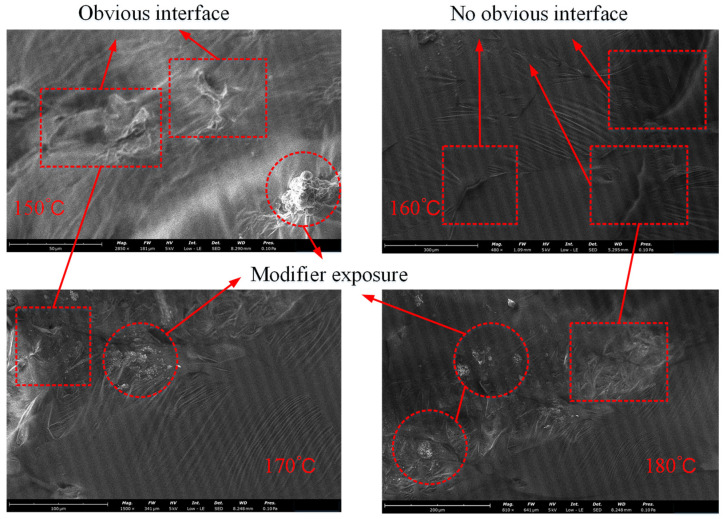
SEM test results of modified asphalt (Magnifications are: 150 °C, ×2850, 160 °C, ×480, 170 °C, ×1500, 180 °C, ×810).

Scanning electron microscopy (SEM) imaging involves the stimulation of electron beams and the collection of physical signals from the surface of the sample under investigation, reflecting its surface topography. For the analysis of SBR-modified asphalt samples prepared at various temperatures, these samples were mounted on slides and coated with gold prior to imaging. The EM-30 Plus SEM, produced by COXEM Ltd., (Seoul, Republic of Korea) was employed for this analysis. This instrument facilitated the characterization of the blending of the SBR modifier with matrix asphalt, enabling the examination of the morphology of SBR-modified asphalt prepared at different temperatures, as illustrated in Figure 8.

Based on the observations in Figure 8, the interface characteristics between the SBR modifier and asphalt vary at different preparation temperatures. At a preparation temperature of 150 °C, the SBR modifier is evident on the asphalt surface, resulting in a distinct interface effect. In contrast, at 160 °C, the SBR modifier and asphalt exhibit a uniform distribution, with the interface effect being less pronounced. However, at 170 °C and 180 °C, despite the uniform distribution between the SBR modifier and asphalt, the interface effect is more noticeable compared to 150 °C, suggesting a more optimized distribution of the SBR modifier. Therefore, taking into account the various preparation temperatures, the compatibility between the SBR modifier and asphalt is optimal at 160 °C. This observation aligns with the conclusion drawn from molecular dynamics simulations, further validating the effectiveness of molecular dynamics simulations in investigating the compatibility of SBR modifiers and matrix asphalt.

### 3.3. Dynamic Shear Rheology Based Compatibility Testing

Multiple approaches exist for investigating the compatibility between polymers. The upper section relies on the electron microscope method to assess the compatibility between SBR and asphalt from a microscopic perspective. Conversely, from a macroscopic standpoint, compatibility can be determined using the rheological test method. The Cole–Cole plot, derived from the frequency scan, is employed to evaluate the compatibility of the polymer blend system.

**Figure 9:**
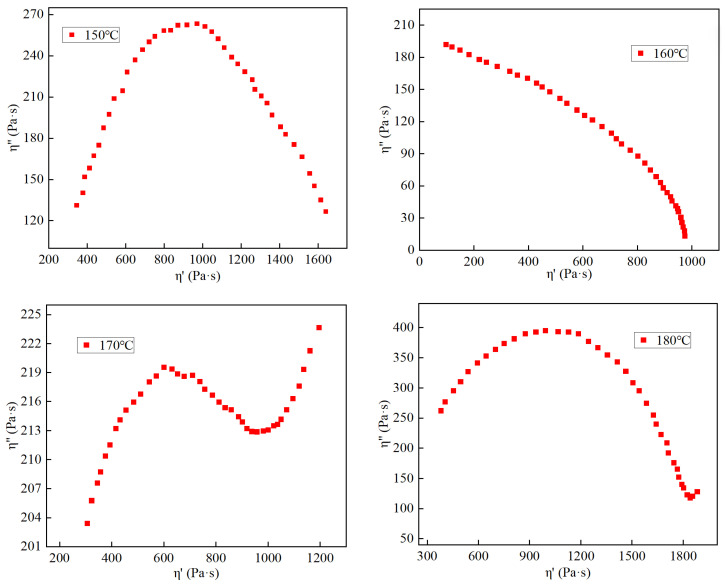
Cole–Cole diagram of SBR-modified asphalt.

The so-called Cole–Cole diagram [27] collects the η′ value (η′ = G″/ω) and η″ value (η″ = G′/ω) relationship diagram. The experimental temperatures were also selected as 160 °C, 170 °C, 180 °C, and 190 °C, and the frequency scanning range was 0.01–100 Hz. Generally speaking, for a polymer blend system with a unimodal molecular weight distribution, the relationship curve between the real part η′ and the imaginary part η″ of the complex viscosity is a half arc, which has a good compatibility; while for incompatible polymers for the blending system, the relationship curve will form a characteristic that deviates from the semi-circular arc. The Cole–Cole diagram of the SBR-modified asphalt at different temperatures is shown in Figure 9.

The observations from Figure 9 indicate that the Cole–Cole plots of all modified asphalt samples display a circular shape, implying compatibility between asphalt and SBR. However, it is important to note that only the curve corresponding to 160 °C maintains a consistently circular shape, with deviations or steepening observed at other temperatures. Interpreting the Cole–Cole plot to evaluate the compatibility of SBR and asphalt, it can be deduced that the optimal compatibility between asphalt and SBR is achieved at 160 °C. Furthermore, it can be hypothesized that the modified asphalt prepared at this temperature may exhibit superior rheological properties.

## 4. Conclusions

(1) Through the examination of the SBR-modified asphalt blending model, it was discovered that at 160 °C, the solubility parameter difference between the SBR modifier and matrix asphalt was minimal, and the interaction was at its strongest, indicating optimal compatibility. The interactions within the SBR-modified asphalt blending systems were primarily governed by van der Waals forces, and the addition of SBR improved the organization of the asphalt molecules.
(2) The SEM test outcomes revealed that the compatibility interface between SBR and asphalt was the most stable at 160 °C. Additionally, based on the Cole–Cole plot, the rheological properties of the modified asphalt prepared at this temperature were deemed to be superior.

## Figures and Tables

**Table 1:** Parameters of four components of matrix asphalt.

Components	Molecular Formula	Molecular Number	Percentage of Components (%)
Asphaltene	C_51_H_62_S	2	10.35
	C_42_H_54_O	2	
	C_66_H_81_N	1	
Saturation	C_35_H_62_	4	10.86
	C_30_H_62_	4	
Aromatic	C_30_H_46_	21	50.68
	C_35_H_44_	18	
Resin	C_18_H_10_S_2_	8	28.11
	C_36_H_57_N	4	
	C_29_H_50_O	4	
	C_40_H_60_S	3	
	C_40_H_59_N	3	

**Table 2:** Composition proportions of SBR.

Component	Mass Ratio	Molecular Weight	Number of Molecules
Styrene	14.8%	106 g/mol	2
Trans butadiene	62.6%	56 g/mol	9
Cis butadiene	7.8%	56 g/mol	1
1.2 Butadiene	14.8%	56 g/mol	2

**Table 3:** Energy of the SBR co-mingled model at different temperatures (kJ/mol).

Temperature (°C)		140	150	160	170	180	190
SBR-modified asphalt	E_p_	45,548.37	45,851.15	46,775.12	47,576.17	49,158.31	49,519.14
	E_v_	−2591.69	−2509.28	−2432.36	−2253.12	−2248.35	−2118.20
	E_e_	−3756.62	−3778.64	−3778.18	−3745.41	−3755.66	−3774.83
Matrix asphalt	E_p_	45,360.09	45,780.30	46,767.42	47,414.54	49,007.48	49,390.74
	E_v_	−1807.38	−1732.21	−1522.42	−1474.92	−1438.47	−1292.54
	E_e_	−3744.91	−3776.96	−3758.88	−3736.16	−3747.79	−3766.37
SBR	E_p_	969.37	1023.99	1098.60	1147.11	1158.53	1165.19
	E_v_	119.77	131.91	137.73	146.39	159.45	171.38
	E_e_	−9.17	−2.72	−14.27	−5.40	−7.20	−6.36

**Table 4:** Technical specifications of matrix asphalt.

Technical Indicators	Density (g/cm^3^)	Penetration at 25 °C (0.1 mm)	Ductility at 15 °C (mm)	Softening Point (°C)	Flash Point (°C)
Numerical value	1.000	68	>100	49.1	>220

**Table 5:** Technical specifications of the SBR modifier.

Density (g/cm^3^)	Constant Tensile Strength (100%)	Tearing Strength	Friction	Hardness	S/B	Copolymer Type
0.94	6.0 MPa	15.1 MPa	0.46	77	0.3–0.35	Random copolymer

## Data Availability

Data are contained within the article.
